# Psoriasis in Saudi Population: Gender Differences in Clinical Characteristics and Quality of Life

**DOI:** 10.7759/cureus.22892

**Published:** 2022-03-06

**Authors:** Awadh Alamri, Raneem Alqahtani, Ibrahim Alshareef, Amjad Alshehri, Atheel Balkhy

**Affiliations:** 1 Dermatology, King Abdulaziz Medical City, Jeddah, SAU; 2 Medicine, College of Medicine, King Saud Bin Abdulaziz University for Health Sciences, Jeddah, SAU; 3 Dermatology, College of Medicine, King Saud Bin Abdulaziz University for Health Sciences, Jeddah, SAU

**Keywords:** clinical characteristics, gender roles, psoriasis, quality of life (qol), saudi population

## Abstract

Introduction

Patients with psoriasis suffer from an inflammatory immune-mediated skin condition that impacts their quality of life severely. In our study, we aimed to analyze the gender differences in clinical characteristics in patients with psoriasis and to assess the quality of life in these patients.

Methods

This is a retrospective observational study that was conducted at King Abdulaziz Medical City (KAMC) in Jeddah, Saudi Arabia.

Results

A total of 139 patients with psoriasis met the inclusion criteria, with a mean ± SD age of 45.53 ± 15.3 years. No statistically significant gender differences were observed in terms of disease duration, BMI, clinical type and body surface area (BSA) (P=0.657, P=0.782, P=0.565, P=0.088, respectively). However, psoriasis caused a significant impairment in the quality of life in female patients compared to males (P=0.036).

Conclusion

This study reports the gender differences in clinical characteristics in patients with psoriasis and its impact on the quality of life. More researches with larger sample size and different populations are needed in order to identify more potential gender-specific variations.

## Introduction

Psoriasis is a chronic immune-mediated skin and joint disease that can present at any age and in numerous clinical forms, in which plaque psoriasis is the most prevalent form among others. The shape, location, and severity of psoriasis can be extremely diverse. Furthermore, psoriasis can take a variety of forms, including chronic, stable plaques or an abrupt onset with rapid advancement and widespread involvement. The exact etiology is still unknown but multiple genetic and environmental factors play a role [1-2].

Psoriasis is not only a disease of the skin and joints. Several previous studies have found that different medical comorbidities have been associated with psoriasis [1,3]. Although psoriasis has no influence on mortality, it does have a variety of substantial negative consequences for patients, as evidenced by a significant reduction in quality of life (QoL). Moreover, it has been reported that patients with psoriasis have a significant prevalence of psychological illnesses or distress [2,4-6].

Psoriasis has notable differences between men and women in different clinical variables, such as disease severity, comorbidities and the use and compliance with treatment. Anatomical skin changes, genetic differences, social and cultural influences contribute to these differences [7]. Previous researchers have studied the gender differences in various aspects of psoriasis. In a study done in Korea, male patients are more likely than female patients to experience moderate to severe disease activity (56.1% vs 51.7%) [8]. Similarly, in Nordic, psoriasis treatment differs significantly by gender, as a higher proportion of females used topical treatment and a greater proportion of males used systemic treatments [9].

The aim of the present observational study was to analyze the gender differences in clinical characteristics in patients with mild to severe psoriasis and to assess the impact of quality of life in these patients.

## Materials and methods

Study setting and participants

This is a retrospective, observational study that was conducted at King Abdulaziz Medical City (KAMC) in Jeddah, Western Region, Saudi Arabia. All patients with psoriasis that were examined and diagnosed by dermatologists from January 2017 to December 2021 were included in the study. Patients with missing information were excluded from the study.

Data collection

Using a self-administered data collection sheet, we reviewed the medical records of the patients who met the inclusion criteria. Data including patient’s demographic information along with medical comorbidities, BMI, disease duration, clinical type were collected. Moreover, the severity of psoriasis was assessed by using Body Surface Area (BSA) score which was then categorized as mild (<3%), moderate psoriasis (3-10%), and severe psoriasis (>10%). Dermatology Life Quality Index (DLQI) score was used to assess the impact of the skin disease on the patient’s quality of life.

Statistics and data analysis

Data were analyzed using SPSS (Statistical Package for the Social Sciences) version 26 (IBM Corp., Armonk, NY, USA). Information and data were saved digitally and only accessed by the investigators. Continuous data are presented as a mean and standard deviation, and categorical variables as frequency and percentage. A p-value < 0.05 was considered significant. To compare categorical variables, Chi-Square test was used and Mann-Whitney U-test was used for continuous variables.

Ethics

Approval from the Regional Research and Ethics Committee at King Abdullah International Medical Research Center Western Region (NRJ21J/285/11) was obtained and ethical considerations were taken throughout the research steps.

## Results

During the study period, a total of 139 patients with psoriasis met the inclusion criteria. Of these participants, 79 (56.8%) were male and 60 (43.2%) were female, with a mean ± SD age of 45.53 ± 15.3 years. There were no statistically significant differences observed between males and females regarding disease duration as the majority of patients had the disease for longer than 10 years (P=0.657). Moreover, there was no significant difference between men and women in terms of Body Mass Index (BMI) (P=0.782). Most of the patients had plaque psoriasis (48.2%), followed by those who had unidentified psoriasis (25.9%), psoriatic arthritis was noted in 12.2% of patients, guttate psoriasis in 7% of patients. There were few cases of inverse psoriasis in 5%, pustular psoriasis in 2.9%, and nail psoriasis with no cutaneous lesion was observed in 2.2% of patients. Nevertheless, there was no significant difference between both genders in the clinical type of psoriasis (P=0.565). In addition, no statistically significant difference in Body Surface Area (BSA) assessment was seen, as the majority of patients in both genders had moderate psoriasis (P=0.088). The differences in the clinical variables of psoriasis between men and women are shown in Table 1.

**Table 1:** Differences in the Clinical Variables between Men and Women in Patients with Psoriasis BSA: Body Surface Area

Disease duration (years), n (%)	All (n = 139)	Male (n = 79)	Female (n = 60)	P
< 10	39 (28.1)	21 (26.6)	18 (30.0)	0.657
≥ 10	100 (71.9)	85 (73.4)	42 (70.0)	
BMI				
< 30	63 (45.3)	35 (44.3)	28 (46.7)	0.782
≥ 30	76 (54.7)	44 (55.7)	32 (53.3)	
Clinical Type, n (%)				
Plaque psoriasis	67 (48.2)	41 (51.9)	26 (43.3)	0.565
Unidentified psoriasis	36 (25.9)	23 (29.1)	13 (21.7)	
Psoriatic arthritis	17 (12.2)	7 (8.9)	10 (16.7)	
Guttate psoriasis	7 (5)	3 (3.8)	4 (6.7)	
Inverse psoriasis	5 (3.6)	2 (2.5)	3 (5)	
Pustular psoriasis	4 (2.9)	2 (2.5)	2 (3.3)	
Nail Psoriasis	3 (2.2)	1 (1.3)	2 (3.3)	
BSA, n (%)				
Mild (< 3%)	20 (14.4)	7 (8.9)	13 (21.7)	0.088
Moderate (3-10%)	93 (66.9)	55 (69.6)	35 (63.3)	
Severe (>10%)	26 (18.7)	17 (21.5)	9 (15)	

Dermatology Life Quality Index (DLQI) score was used to evaluate the impact of psoriasis on the patient’s quality of life. In this study, 25% of the female patients experienced an extremely large effect on their quality of life (P= 0.036). A summary of the differences in the quality of life in patients with psoriasis between men and women is shown in Table 2.

**Table 2:** Differences in the Quality of Life in Patients with Psoriasis between Men and Women DLQI: Dermatology Life Quality Index

DLQI, n (%)	All (n = 139)	Male (n = 79)	Female (n = 60)	P
No to small effect	21 (15.1)	13 (16.5)	8 (13.3)	0.036
Moderate to very large effect	96 (69.1)	59 (64.4)	37 (61.7)	
Extremely large effect	22 (15.8)	7 (8.9)	15 (25)	

## Discussion

This present study aimed to identify the differences in clinical characteristics and the impact of psoriasis on the quality of life between men and women in patients treated at KAMC. We observed that male patients accounted for 56.8% and female patients for 43.2%, with a mean ± SD age of 45.53 ± 15.3 years. In a study conducted in Spain, the mean age was 42.34 (median 42.00, interquartile range 25.00-59.00 years) and the percentage of males was 50.66% (201,977 males) [10]. Moreover, a male predominance was observed in multiple other researches [11-12]. In our study, the disease duration has no statistically significant difference (P=0.657). Similar to our findings, Napolitano et al. found no significant difference in terms of disease duration between both genders (P=0.37) [13]. On the contrary, a study by Hägg et al. found that disease duration was longer for women compared with men (P=0.002) [14]. In a study done in Brazil, females with psoriasis had a higher risk for central dyslipidemia than males with the disease [15]. Moreover, a study in Sweden also found that women with psoriasis had a significantly stronger association with obesity than men [16]. Nevertheless, in our study, no significant difference between men and women in terms of BMI was found (P=0.782).

In this study, there was no significant difference between males and females in terms of clinical type. Plaque psoriasis was found to be the most common phenotype among both sexes. However, in a study done by Napolitano et al., female sex was significantly associated with psoriasis clinical type other than diffuse plaque psoriasis such as palmoplantar, guttate, arthro-pathic, pustular and localized plaque types (P=0.0001). Moreover, a palmoplantar pustulosis was found in a previous study to be predominant in female patients [17]. Furthermore, a number of studies have observed that generalized pustular psoriasis (GPP) is predominantly found in females in comparison to males [18-20].

There are numerous measures to assess the clinical severity of psoriasis, such as Body Surface Area (BSA), Psoriasis Area and Severity Index (PASI), Psoriasis Log-based Area and Severity Index (PLASI), Physician Global Assessment (PGA). In the current study, there was no statically significant sex-specific difference in BSA (P=0.088). In agreement, a study in Italy found that there was no difference in PASI between psoriasis patients with moderate to severe psoriasis based on gender [21]. However, the study conducted by Napolitano et al. found that a higher PGA score and PASI ≥ 10 was associated with male sex. Moreover, the study done by Hägg et al. found that the median PASI was higher in men than women. Also, a study in Sweden showed that male patients had a higher PASI compared to females [22].

In the present study, psoriasis caused an extremely large effect on the quality of life in 25% of female patients (P= 0.036). Moreover, in a study done on hospitalized patients with psoriasis in Italy, it showed that older women had the greatest impairment in quality of life [6]. In addition, the study performed by Napolitano et al. noted that female patients had a higher Skindex-17 score, which indicates that psoriasis negatively impacts their quality of life more. Moreover, a Swedish study found that women have a significantly lower health-related quality of life (HRQoL) compared to men [22]. According to a Greek study, DLQI differences among men and women were not statistically significant, but female patients reported lower self-esteem than men [23].

This study aimed to assess the gender differences in clinical variables and the impact of psoriasis on the quality of life. Nevertheless, our study has some limitations. First, the sample is not considered large enough to generalize the results. Second, the research design is retrospective in nature. Lastly, the lack of screening and assessment measures.

## Conclusions

Psoriasis is one of the most prevalent dermatological diseases and is associated with negative impact on the quality of life. There are limited studies in Saudi Arabia that analyzed the gender differences in clinical characteristics and quality of life in patients with psoriasis. In this study, there was no statistically significant difference between the gender in terms of disease duration, BMI, clinical type and BSA. Nevertheless, psoriasis caused a significant impairment on the quality of life in female patients compared to males. Overall, more researches with larger sample size and different populations are needed in order to identify more potential gender-specific variations.
