# Correction: Psoriasis in Saudi Population: Gender Differences in Clinical Characteristics and Quality of Life

**DOI:** 10.7759/cureus.c393

**Published:** 2026-01-09

**Authors:** Awadh Alamri, Raneem Alqahtani, Ibrahim Alshareef, Amjad Alshehri, Atheel Balkhy

**Affiliations:** 1 Dermatology, King Abdulaziz Medical City, Jeddah, SAU; 2 Medicine, College of Medicine, King Saud Bin Abdulaziz University for Health Sciences, Jeddah, SAU; 3 Dermatology, College of Medicine, King Saud Bin Abdulaziz University for Health Sciences, Jeddah, SAU

This article has been updated to add an additional affiliation for Awadh Alamri.

